# Human milk fungi: environmental determinants and inter-kingdom associations with milk bacteria in the CHILD Cohort Study

**DOI:** 10.1186/s12866-020-01829-0

**Published:** 2020-06-05

**Authors:** Shirin Moossavi, Kelsey Fehr, Hooman Derakhshani, Hind Sbihi, Bianca Robertson, Lars Bode, Jeffrey Brook, Stuart E. Turvey, Theo J. Moraes, Allan B. Becker, Piushkumar J. Mandhane, Malcolm R. Sears, Ehsan Khafipour, Padmaja Subbarao, Meghan B. Azad

**Affiliations:** 1 Department of Medical Microbiology and Infectious Diseases, University of Manitoba, Winnipeg, MB Canada; 2 Children’s Hospital Research Institute of Manitoba, Developmental Origins of Chronic Diseases in Children Network (DEVOTION), Winnipeg, MB Canada; 3 Digestive Oncology Research Center, Digestive Disease Research Institute, Tehran University of Medical Sciences, Tehran, Iran; 4 Department of Medicine, McMaster University, Hamilton, ON Canada; 5 Department of Pediatrics, University of British Columbia, Vancouver, BC Canada; 6 Department of Pediatrics and Larsson-Rosenquist Foundation Mother-Milk-Infant Center of Research Excellence (MOMI CORE), University of California San Diego, La Jolla, CA USA; 7 Dalla Lana School of Public Health, University of Toronto, Toronto, ON Canada; 8 Division of Respiratory Medicine, Department of Pediatrics, Hospital for Sick Children, University of Toronto, Toronto, ON Canada; 9 Department of Pediatrics and Child Health, University of Manitoba, Winnipeg, MB Canada; 10 Department of Pediatrics, University of Alberta, Edmonton, AB Canada; 11 Department of Animal Science, University of Manitoba, Winnipeg, MB Canada; 12 Department of Physiology, University of Toronto, Toronto, ON Canada

**Keywords:** Breastmilk, Human milk, Breastfeeding, Mycobiota, Fungi, Environment, CHILD cohort study

## Abstract

**Background:**

Fungi constitute an important yet frequently neglected component of the human microbiota with a possible role in health and disease. Fungi and bacteria colonise the infant gastrointestinal tract in parallel, yet most infant microbiome studies have ignored fungi. Milk is a source of diverse and viable bacteria, but few studies have assessed the diversity of fungi in human milk.

**Results:**

Here we profiled mycobiota in milk from 271 mothers in the CHILD birth cohort and detected fungi in 58 (21.4%). Samples containing detectable fungi were dominated by *Candida*, *Alternaria*, and *Rhodotorula*, and had lower concentrations of two human milk oligosaccharides (disialyllacto-N-tetraose and lacto-N-hexaose). The presence of milk fungi was associated with multiple outdoor environmental features (city, population density, and season), maternal atopy, and early-life antibiotic exposure. In addition, despite a strong positive correlation between bacterial and fungal richness, there was a co-exclusion pattern between the most abundant fungus (*Candida*) and most of the core bacterial genera.

**Conclusion:**

We profiled human milk mycobiota in a well-characterised cohort of mother-infant dyads and provide evidence of possible host-environment interactions in fungal inoculation. Further research is required to establish the role of breastfeeding in delivering fungi to the developing infant, and to assess the health impact of the milk microbiota in its entirety, including both bacterial and fungal components.

## Background

Fungi constitute an important yet frequently neglected component of the human microbiome [1, 2] with a possible role in health and disease [3, 4]. Fungal and bacterial colonisation occur in parallel during early life [5], yet most infant microbiome studies have overlooked fungi. Milk is a source of diverse and viable bacteria [6], but only a few studies have assessed fungi in human milk [7–12]. Additionally, although geographical differences were observed in a recent study of 80 women from 4 countries [9], the maternal, infant, and environmental determinants of milk fungi (mycobiota) are still mostly unknown.

Bovine and human studies have confirmed the presence of potentially viable fungi in milk using microscopy, culture-dependent, and culture–independent methods [7–10, 13]. One metagenomics study of human milk suggests that fungi likely constitute 0.5–2% of the milk microbial community [8]. Another study of 76 mother-infant dyads found that maternal age, blood type, antibiotics, vaginal delivery, and infant sex were associated with *Candida* colonisation of the infant, and in a subset, maternal vaginal and rectal samples were identified as potential origins of this taxon [14]. Bottle feeding and frequent pacifier use was associated with a higher rate of oral *Candida* carriage in asymptomatic infants [15, 16]. Host genetic variation in major histocompatibility genes were also associated with variations in bovine milk mycobiota [13]. Home characteristics and season are associated with indoor fungi [17, 18] and thus could plausibly influence milk mycobiota; however, mothers’ milk and the home environment (another potential source of milk fungi) have not been examined.

Bacteria-fungi interactions are ubiquitous in microbial communities including the human gut microbiota. These interactions occur through direct physical interactions or several types of molecular communications affecting the composition and function of each respective assemblage [19]. Both bacteria and fungi compositions are altered in different disease conditions including inflammatory bowel disease [20, 21]. Inter-kingdom bacteria-fungi interactions have rarely been investigated in milk [9, 13]. To address these open questions, we analyzed the presence, composition, and determinants of human milk mycobiota at 3–4 months postpartum in a subset of Canadian mother-infant dyads from the CHILD Cohort Study [22].

## Results

In our study population (Table S1) the majority of dyads were urban residents (96%) although population densities ranged widely from 0 to 36,170 persons/km^2^ (median 3447). 67% of mothers were atopic and 25% delivered by Caesarean section. 18% of infants developed possible or probable asthma by the age of 3 years. Mean ± SD bacterial richness and diversity of milk were 145 ± 46 and 15.2 ± 9.2, respectively (Table S2).

### Presence of milk fungi was significantly and independently associated with environmental characteristics, human milk oligosaccharides, and milk bacterial composition

**Fig. 1:**
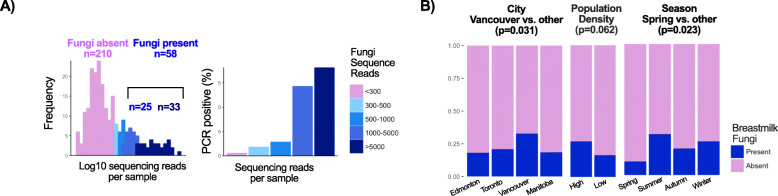
Presence of fungi in human milk and associated environmental, maternal and infant characteristics. **a** Defining fungi presence according to the depth of sequencing informed by the positive PCR bands. **b** Presence of fungi according to study site, population density, and season tested by chi-square test

**Fig. 2:**
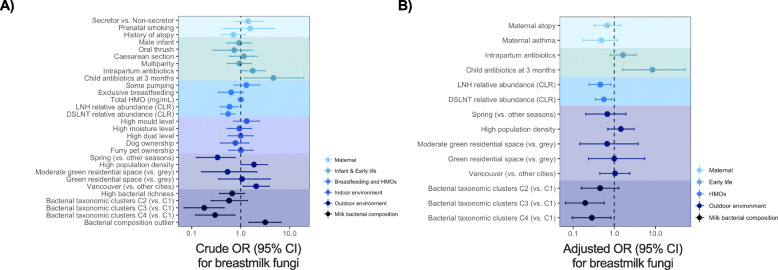
Association of human milk fungi presence with environmental, maternal and infant characteristics. **a** Unadjusted univariate associations of fungal presence with maternal, infant and early life factors, breastfeeding and selected HMOs, indoor and outdoor environmental factors, and milk bacterial composition tested by logistic regression. **b** Adjusted multivariable associations of host and environmental factors with the presence of fungi. Variable selected by LASSO (see Fig. 3c) were included in the multivariable logistic regression

Using a minimum threshold of 1000 reads/sample informed by positive PCR results (Fig. 1a), 58/271 (21.4%) of mothers’ milk samples contained fungi. To address whether milk fungi were more likely acquired externally (e.g. from the environment, maternal skin and/or the infant oral cavity) or internally from the maternal gastrointestinal tract, we assessed associations of fungi presence with environmental vs. maternal or other factors. Both environmental (e.g. study city, population density, and season) and maternal characteristics (e.g. antibiotics and atopy) were associated with fungi presence in univariate analyses (Figs. 1b & 2a). Milk from mothers in Vancouver had the highest presence of fungi (32% vs. 18–21% in Edmonton, Toronto, and Manitoba; unadjusted OR = 2.06, 95%CI 1.11–3.82, for Vancouver vs. other cities; *p* = 0.021; Figs. 1b & 2a). Vancouver is a large coastal Canadian city with more humid climate, higher annual precipitation, and milder winters compared with the other study sites (Table S3), and thus likely has higher load of environmental fungi [23]. In line with this, season and population density were also associated with fungal presence, with the lowest presence detected in spring and at low population density (Figs. 1b & 2a).

Among the maternal, infant, and early life factors assessed, maternal intrapartum antibiotics and infant antibiotics at the time of sample collection were associated with a higher likelihood of fungi presence (Fig. 2a) indicating that bacteria originating from the mother or infant might be associated with the presence of milk fungi. Indeed, milk bacterial taxonomic clusters, defined previously on the basis of hierarchical clustering of core taxa [24], were significantly associated with presence of fungi (*p* < 0.001; Figs. 2a & 4a). Cluster 1 (enriched in *Moraxellaceae*, *Enterobacteriaceae*, and *Pseudomonadaceae*) had the highest presence of fungi (42%) followed by Cluster 2 (enriched in *Streptococcaceae*, *Staphylococcaceae*, and *Oxalobacteraceae*) (30%) compared to Cluster 3 (enriched in *Oxalobacteriaceae* and *Comamonadaceae*) (18%) and Cluster 4 (*Streptococcaceae* and *Comamonadaceae*) (12%).

**Fig. 3:**
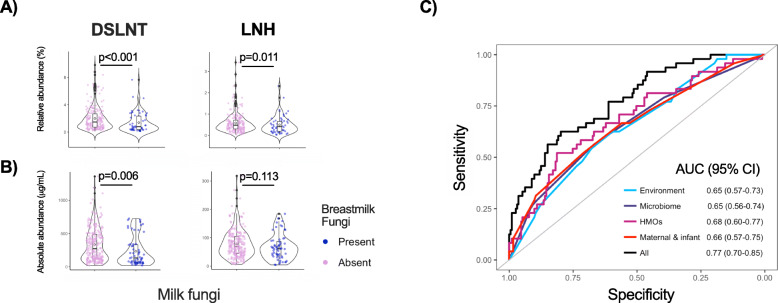
Association of human milk fungi presence with milk components and other factors. **a** Relative abundances and **b** Absolute concentrations of Disialyllacto-N-tetraose (DSLNT) and Lacto-N-hexaose (LNH) based on milk fungi presence tested by ANOVA. **c** Prediction accuracy of fungi presence based on unsupervised variable selection by LASSO. The selected variables were classified as: environmental (study site, population density, season, residential vegetation); bacterial (bacterial taxonomic cluster and bacterial composition outlier); HMOs (DSLNT and LNH); maternal and infant (intrapartum antibiotics, child antibiotics at 3 months, maternal asthma, and maternal atopy)

We also assessed the association of milk fungi with secretor status and the 19 most abundant human milk oligosaccharides (HMOs), which were previously measured in the same samples [25]. Secretor status is genetically determined by polymorphisms in the fucosyl transferase 2 (*FUT2*) gene that influences the synthesis of fucosylated HMOs [26]. The impact of maternal secretor status [27] and HMOs on infant gastrointestinal bacterial composition is well established [28], but little is known about their impact on milk mycobiota. There is some evidence that certain HMOs can inhibit fungi in vitro [29], but it is also plausible that other HMOs could be metabolised by fungi and support their growth. Here, we found no association between secretor status and milk fungi presence; however, two *FUT2*-independent HMOs (disialyllacto-N-tetraose (DSLNT) and lacto-N-hexaose (LNH)) were less abundant in milk containing fungi (Figs. 2a, 3a and b), suggesting that these HMOs might inhibit or be metabolised by milk fungi. Alternatively, these HMOs might indirectly influence the milk mycobiota via modulating the milk microbiota or fatty acids composition [30].

To identify the determinants of milk fungi while controlling for the above-mentioned factors, we performed unsupervised variable selection by least absolute shrinkage and selection operator (LASSO) and used the LASSO-selected variables to predict the presence of milk fungi. In agreement with the logistic regression results above, LASSO identified season, population density, study city, residential vegetation, bacterial clusters, bacterial outliers (previously identified based on variance in the first principal component score [31]), DSLNT, LNH, intrapartum antibiotics, and child antibiotics at 3 months as predictors of fungi presence. In addition, LASSO identified maternal asthma and atopy, suggesting that atopic mothers may have distinct skin fungal load and/or composition, which has been observed in other studies [32]. Combined, these selected variables had a relatively good predictive accuracy for presence of fungi (AUC = 0.77, 95% CI 0.70–0.85) (Fig. 3c). In a multivariable logistic regression model adjusted for all of the LASSO-selected factors, DSLNT, LNH, bacterial taxonomic clusters, and child antibiotics at 3 months were the strongest independent predictors of fungi presence (Fig. 2b). As it is plausible that not all fungal genera respond to these variables similarly, we also examined the association of these factors with the presence of specific fungal taxa (see below).

Among other factors we evaluated, fungi presence was not associated with visible mould or moisture levels in the home, older siblings, pet ownership, maternal BMI, or history of infant oral thrush (a fungal infection caused by *Candida* spp.) in the first 3 months of life (Fig. 2a). We lacked information on breast thrush and mastitis, which are also known to be associated with *Candida* spp. in milk [10]. Additionally, although others have found that milk collection using a pump and bottle feeding were associated with increased presence of *Candida* spp. in milk [16], we did not observe an association between fungi presence and mode of breastfeeding (nursing at the breast vs. pumped milk feeding at least once in the preceding 2 weeks) (Fig. 2a). Breastfeeding exclusivity (i.e. formula supplementation) at the time of sample collection was also not associated with fungal presence (Fig. 2a).

### Milk microbiota composition differs in the presence vs. absence of breastmilk fungi

**Fig. 4:**
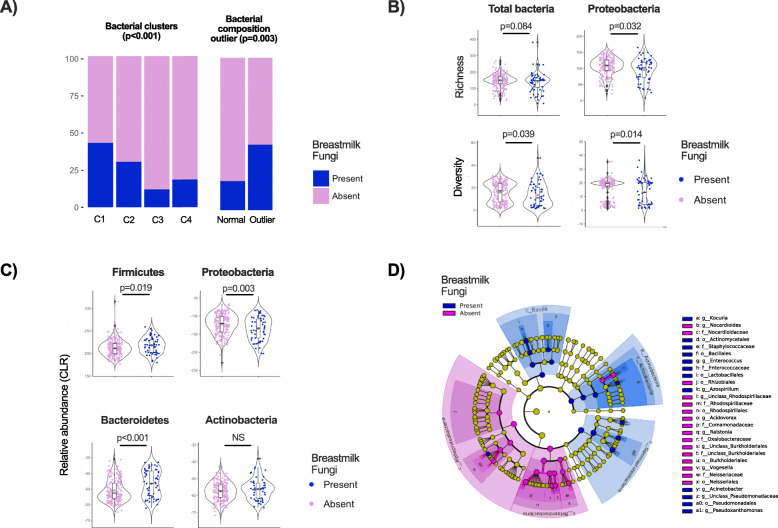
Association of human milk bacteria and fungi. **a** Presence of fungi according to bacterial taxonomic clusters and bacterial outliers. **b** Association of total bacteria and Proteobacteria richness and diversity with presence or absence of milk fungi tested by Wilcoxon rank sum test. **c** Differential abundance of milk major bacterial phyla with presence or absence of milk fungi. **d** Linear discriminant analysis of milk bacteria according to presence or absence of fungi

Since we observed that bacterial taxonomic clusters were associated with fungi presence (Figs. 2a & 4a), we further explored the relationship between milk bacteria and mycobiota. In our previous analysis of milk bacteria, we identified “outlier” samples based on variance in the first principal component score [31] (Figure S1). Outlier status was not associated with any technical or biological variables previously assessed, but here we found that outliers had significantly higher presence of fungi compared to the rest of the samples (43% vs. 19%, OR = 3.17 95%CI 1.49–6.65, *p* = 0.002) (Figs. 2a & 4a). Additionally, Proteobacteria richness and diversity were lower in samples containing fungi (Fig. 4b), and a similar trend was observed for total milk bacteria. Overall, the relative abundance of Proteobacteria was lower while Firmicutes and Bacteroidetes were higher in the presence of fungi (Fig. 4c). Using linear discriminant analysis [33], members of Actinobacteria, Bacilli, and γ-Proteobacteria were enriched in the presence of fungi while α-Proteobacteria and β-Proteobacteria were depleted (Fig. 4d).

### Milk fungi profile was dominated by Basidiomycota and Ascomycota and tended to differ by study site, maternal antibiotic use, and maternal atopy

**Fig. 5:**
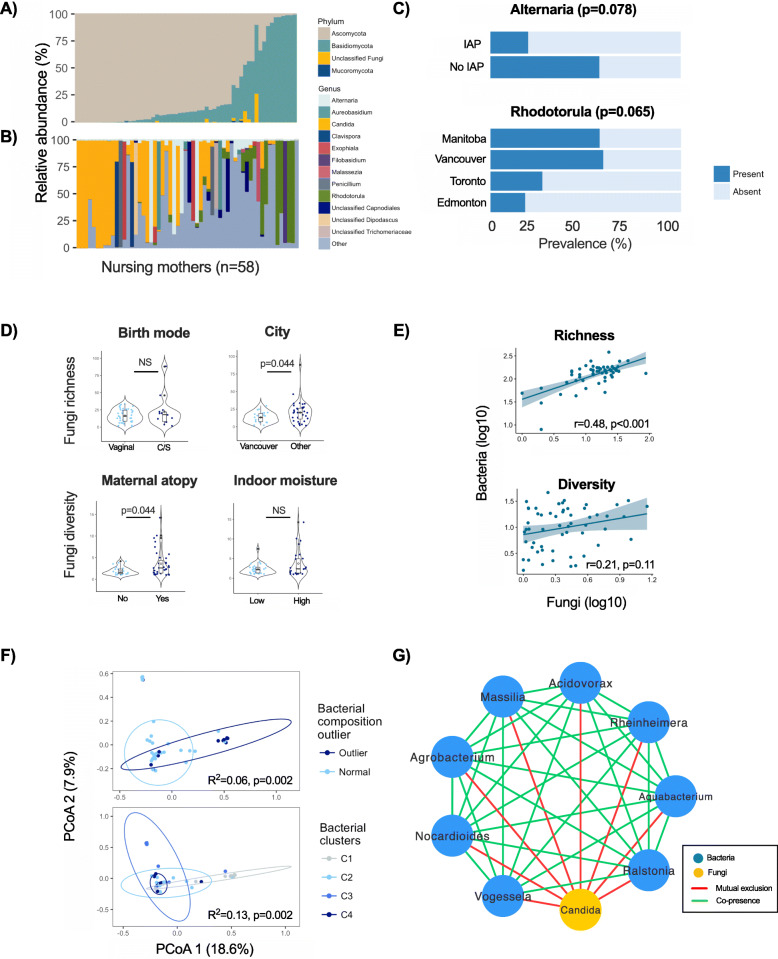
Association of milk mycobiota diversity and composition with environmental and maternal factors and milk bacterial composition. Milk fungi taxonomy at (**a**) phylum and (**b**) genus levels. Samples are in the same order in panels **a** and **b**. **c** Association of intrapartum antibiotics and study city with the presence of selected fungal species tested by chi-squared test. **d** Association of birth mode and city with fungal richness, and of maternal atopy and indoor moisture level with fungal diversity, tested by Wilcoxon rank sum test. **e** Correlation of bacteria and fungi richness and diversity. **f** Association of milk bacterial clusters and bacterial composition outliers with fungal β diversity assessed on Bray-Curtis dissimilarity matrix and tested by PERMANOVA. **g** Co-occurrence analysis of most abundant bacteria and fungi (> 1% mean relative abundance) in milk based on Spearman rank correlation. Only significant edges with r > |0.5| are visualised. Nodes with no connections were removed, including 5 bacterial and 11 fungal taxa. IAP, intrapartum antibiotics. ~ *p* < 0.1, * *p* < 0.05

Next, among the 58 samples with detectable fungi, we assessed fungal composition and diversity. Ascomycota and Basidiomycota were the dominant fungal phyla in the milk (Fig. 5a) in agreement with previous reports [7, 9]. Composition was quite heterogeneous at the genus level (Fig. 5b). Overall, 12 genera had a minimum mean relative abundance of > 1% (Table S4). The most prevalent fungi were *Candida* (present in 60% of samples containing fungi, mean 28.6% ± 38.2%% relative abundance), *Alternaria* (50%, mean 6.9% ± 20.8%), and *Rhodotorula* (43%, mean 10.3% ± 24.7%). Some samples were dominated by one genus (relative abundance > 50%) - most frequently belonging to *Candida* (*n* = 17, 29% of samples), *Alternaria* (*n* = 4, 7%), or *Rhodotorula* (*n* = 6, 10%). Less prevalent yet dominant genera within some samples included *Clavispora* (*n* = 2, 3%), *Exophiala* (n = 2, 3%), and *Penicillium* (n = 2, 3%). Other samples contained multiple less prevalent genera (Fig. 5b). Acknowledging the low power due to small sample size, we detected borderline differences in the presence of *Alternaria* according to intrapartum antibiotics exposure, and *Rhodotorula* between study cities (Fig. 5c).

Mean fungal richness and diversity at amplicon sequencing variant (ASV) level were 17.9 ± 13.7 and 2.9 ± 2.6, respectively. Fungal diversity was higher in atopic vs. non-atopic mothers (3.6 vs. 1.9, *p* = 0.044) (Fig. 5d). We did not find any other significant associations between fungal taxonomic structure or diversity with other maternal, infant, early life, environmental, and milk factors (not shown).

### Milk fungi exhibit co-exclusion associations with milk bacteria

Finally, we assessed the inter-kingdom associations of abundant milk bacteria and fungi (taxa with > 1% mean relative abundance). Interestingly, bacterial richness was positively correlated with fungal richness (both assessed at the ASV level, *r* = 0.48, *p* < 0.001; Fig. 5e) though bacterial and fungal diversities were not significantly correlated (*r* = 0.21, *p* = 0.11). In addition, bacterial taxonomic clusters and compositional outliers were significantly associated with fungi α (Figure S2) and β diversity (Fig. 5f). While several bacterial genera were positively associated with each other, indicative of co-presence, we did not detect any associations between different fungal genera. *Candida* was the only fungal genus associated with bacterial genera; these inter-kingdom associations were negative, suggesting a mutual exclusion relationship (Fig. 5g).

## Discussion

We found that approximately 20% of milk samples contained detectable fungi and provide evidence that fungal presence was associated with environmental characteristics and milk bacteria. Milk fungal taxonomy was consistent with previous reports [7, 9]. Ascomycota and Basidiomycota were the dominant fungal phyla in the milk and three genera of *Candida*, *Alternaria*, and *Rhodotorula* were the most prevalent taxa. Some of the previously reported fungal genera including *Saccharomyces* and *Aspergillus* [7, 9] were not among the abundant genera identified in our study, perhaps due to geographical variations and/or other differences in the participants characteristics. To our knowledge, this is one of the largest studies of human milk mycobiota performed to date, and the only milk mycobiota study to examine home environmental characteristics. Our results expand considerably upon existing knowledge about milk fungi, providing evidence for bacterial-fungal interactions within the human milk microbiome.

### Are fungi universally present in milk?

It is intriguing that fungi were not present in the majority of samples in our study. Previous culture-dependent and -independent studies in bovine animals [13, 34] and humans [7–10, 16] have confirmed the presence of fungi in milk. However, while the majority of bovine milk samples contained detectable levels of fungi [13], the presence of fungi in human milk has varied from 40% (by culture), to 35–80% (qPCR), and 70–100% (sequencing) in different studies [7, 9, 11]. The presence of fungi in our study was lower than previously reported sequencing estimates; potentially due to the higher and more conservative threshold we applied to define fungal presence, other methodological differences (e.g. milk sample collection method, the volume of milk used for DNA extraction, sequencing depth, or target region: ITS2 in our study and 28S rRNA and ITS1 in previous reports), true geographical variations among countries (Canada in our study vs. Spain, South Africa, Finland, and China in previous reports), and/or differences between study cohorts, sampling time, and mode of breastfeeding. Our results are consistent with reports of non-universal fungal presence in the infant gastrointestinal tract over the first year of life [4]. Given that fungi constitute the rare biosphere of the human microbiota [1], it is conceivable that a greater sampling effort may improve their identification in low biomass samples such as milk.

### Origins and maternal determinants of milk fungi

The origins of milk fungi are unclear and it is not known whether milk is evolved to transfer maternal fungi to the infant. The external (retrograde inoculation) and internal (oro-entero-mammary) pathways hypothesized for the origins of milk bacteria [35, 36] could potentially be extended to milk fungi. However, the supporting evidence in general is lacking. Additionally, it is not clear whether fungi exist in the intramammary milk or only following expression potentially originating from maternal skin. Dominance of milk mycobiota by *Candida* (a common oral fungi [37]) in a subset of samples in our study and others [7, 10, 11] suggest the infant oral cavity as a potential source of milk fungi even in the absence of symptomatic oral thrush [15], while dominance of environmental fungi such as *Penicillium*, *Exophiala*, and *Rhodotorula* in other samples suggest contribution from maternal skin or other environmental surfaces.

Although previous studies have found that milk collection using a pump, bottle feeding, and frequent pacifier use were associated with increased presence of *Candida* spp. in human milk and the infant oral cavity [15, 16], we did not observe any differences in mycobiota presence, diversity, or composition according to mode of breastfeeding (nursing at the breast vs. pumped and bottle feeding at least once in the preceding 2 weeks). It remains possible that pumping has a transient effect on the milk mycobiota that was not detectable in our study.

Our findings suggest the diversity of milk fungi is influenced by maternal characteristics, such as atopy. The direction of association with maternal atopy warrants further investigation, as it is possible that host immunity influences both atopic sensitization and fungal colonization of the skin and/or milk, or alternatively, that environmental fungi influence both maternal atopy and milk mycobiota [32]. Additionally, intrapartum antibiotic exposure was associated with lower presence of *Alternaria* in milk. Although antibiotics do not directly target fungi, their impact on the overall bacterial composition has been shown to eliminate constraints on fungal (mainly *Candida*) colonisation and growth [38]. It is therefore plausible that intrapartum antibiotics indirectly modulate the fungal community in the maternal vaginal and/or gastrointestinal tract as well as the infant oral cavity – all of which are potential sources of milk fungi.

To our knowledge, ours is the first study to report associations between specific HMOs and milk fungi, which warrants further exploration. There is preliminary evidence that milk protein content might be associated with milk fungi [7], but the extent to which other milk components including HMOs and immunomodulatory factors influence milk fungi remains to be elucidated.

### Regional and seasonal differences in the presence of fungi

The environmental pool of available species is an important factor influencing the formation and composition of microbial communities [39]. Similar to Boix-Amorós et al., we observed geographical variations in the presence of milk fungi with the highest prevalence of fungi observed in milk from mothers residing in Vancouver (a large coastal Canadian city with humid climate, high annual precipitation, and mild winters) compared with smaller Canadian cities in the Prairies with lower average temperature precipitation. This difference was partly explained by differences in population density and season, but was not related to other characteristics of the outdoor environment (e.g. greenness) or home environment (e.g. mould, dust). Presence of fungi was lower in milk samples collected in spring consistent with seasonal variations reported in goat milk fungi [40] which could be partly attributed to fluctuations in outdoor [41] and indoor [42] environmental fungi levels.

### Bacteria-fungi interaction in the milk: direct or indirect interaction?

We observed associations between bacterial composition and presence and relative abundance of fungi, suggesting that some milk bacterial communities were more permissive to fungal presence, or vice-versa. Correlation of bacterial and fungal richness but not diversity suggests that certain factors may impact microbial dispersal in general, and thus enhance both bacterial and fungal richness, whereas the diversity of each kingdom is separately determined by the niche relevant biotic and abiotic factors. To our knowledge, only one previous study has examined the potential interaction of fungi and bacteria in milk, finding a positive correlation between the skin inhabitant genus *Malassezia* and bacterial load [7]. Our results provide preliminary evidence for antagonistic bacteria-fungi interactions, consistent with evidence that some milk bacteria demonstrate antifungal properties [43, 44], as observed in other environments [45]. Overall, these results could reflect one or more of the following biological relationships: a) some milk bacterial communities are more permissive to fungal presence and proliferation, b) milk bacterial dynamics are influenced by fungi when they are present, and/or c) milk bacterial composition is influenced by the milk environment, which also independently facilitates fungal inoculation or colonisation [46]. More research is needed to determine whether this observation is due to active fungi-bacteria ecological interactions within the milk environment, or perhaps the infant oral cavity.

### Strengths and limitations

This study is among the first to assess milk mycobiota in association with home environment characteristics and milk bacterial composition. A key limitation is that we defined the threshold of fungi presence in relation to PCR amplification results. More accurate methods such as quantitative PCR or culture are required to confirm fungi presence. The availability of outdoor and indoor environmental features in addition to host variables allowed us to comprehensively assess determinants of milk mycobiota, although we lacked information on breast thrush (or use of anti-fungal medications) and mastitis, which are known to be associated with *Candida* spp. in milk [10]. Although the infants were generally healthy at the time of sample collection, this study was slightly enriched for infants who developed asthma later in life and this could limit the generalizability to healthy infants. Finally, as with all culture-independent studies, we do not have information on the viability of milk fungi detected in our study.

## Conclusion

**Fig. 6:**
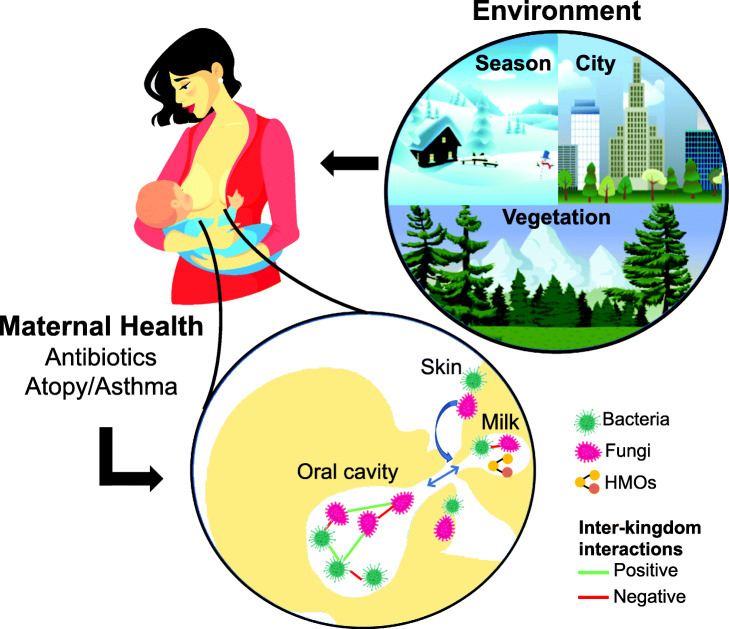
Potential sources of milk mycobiota and factors influencing their presence and composition. Environmental factors including season, city, and vegetation can influence the abundance and composition of the available pool of fungal species that colonise the maternal skin and infant oral cavity. Fungi could be transferred to the milk from the skin and/or infant oral cavity via a retrograde mechanism. Milk bacterial composition could also influence milk fungi either within the milk environment or via interactions on the skin or in the infant oral cavity. Maternal characteristics and components of the milk microenvironment could also influence milk mycobiota

In conclusion, we profiled human milk mycobiota in a well-characterised cohort of mother-infant dyads and provide evidence of possible host-environment interactions in fungal inoculation (Fig. 6). This research on the composition and potential determinants of milk fungi gives rise to several new avenues of exploration. For example, the origins of milk fungi are unclear and it is not known whether milk has evolved to transfer fungi to the infant nasopharyngeal and/or gastrointestinal microbiota. Our data suggest that environmental fungal load is a determinant of milk fungi presence, and that sources of milk fungi likely include the maternal skin and/or infant oral cavity and other environmental surfaces which are more directly influenced by environmental fungi. Further studies are needed to confirm these potential sources and determinants of milk mycobiota. The biological nature of the associations we have observed between bacteria and fungi in milk is also unclear, but could involve competition for nutrient sources or biofilm formation. It is possible that bacteria and fungi form complexes in the infant mouth or maternal skin and are translocated together. The role of milk fungi in infant health and disease should be explored since early life exposure to fungi and gastrointestinal mycobiota have been associated with altered risk of asthma and allergy in children [3, 4, 47, 48]. Further research is required to establish the role of breastfeeding in delivering fungi to the developing infant, and to assess the health impact of the milk microbiota in its entirety, including both bacterial and fungal components. Additionally, further investigation is required to assess the role of milk fungi in infant health outcomes.

## Methods

### Study design

Women with singleton pregnancies were enrolled between 2008 and 2012 into the general population CHILD birth cohort (*n* = 3455), from which a subset of 271 mother-infant dyads was selected for this study [22]. The subset was selected from samples with available 16S rRNA microbiota data [24] and was enriched for children diagnosed with possible or probable asthma. Asthma was diagnosed by an expert study physician at the clinical assessment at age 3 years and was classified for this analysis as “possible or probable asthma” or “no asthma”. Samples with high concentration of DNA based on bacterial 16S rRNA V4 PCR amplification (≥15 ng/μL) were prioritised. Written informed consent was obtained from the participants. The study was approved by the Human Research Ethics Boards at McMaster University, the Hospital for Sick Children, and the Universities of Manitoba, Alberta, and British Columbia.

### Milk sample collection

Each mother provided one sample of milk at 3–4 months postpartum [mean (SD) 17 (5) weeks postpartum] in a sterile milk container provided by the CHILD study. To control for differences in the milk composition of fore- and hindmilk [49] as well as the diurnal variation [50], a mix of foremilk and hindmilk from multiple feeds during a 24-h period was collected. Hand expression was recommended, but pumping was also acceptable. The sample was collected in a real life situation with no recommended hand or environment cleaning procedures. Samples were refrigerated at 4 °C at home for up to 24 h before being collected and processed by study staff [51]. Samples were stored at − 80 °C until analysis.

### DNA extraction and fungal sequencing

DNA was extracted from 1 ml of milk sample as previously described [24]. Milk mycobiota was assessed by sequencing the internal transcribed spacer (ITS) 2 region of the eukaryotic ribosomal gene with modified ITS3/ITS4 primers [13] on a MiSeq platform (Illumina, San Diego, CA, USA) as previously described [13]. For each sample, the PCR reaction was performed in duplicate, consisting of an initial denaturing step at 95 °C for 5 min followed by 32 amplification cycles at 95 °C for 40 s, 52 °C for 45 s, and 72 °C for 40 s, with a final extension step at 72 °C for 5 min in an Eppendorf Mastercycler pro (Eppendorf, Hamburg, Germany). Sterile DNA-free water was used as negative controls in sequencing library preparation. Fecal samples and mock community containing 8 bacteria and 2 fungi species (*Saccharomyces cerevisiae* and *Cryptococcus neoformans*) (Zymo Research, CA, USA) were used as positive controls. PCR amplification was assessed on all samples by agarose gel electrophoresis and visible bands were reported by two independent observations (SM, KF). Duplicate PCR products were pooled and the concentration of double-stranded DNA was quantified by Quanti-iT PicoGreen dsDNA Assay kit (Invitrogen, CA, USA). The concentrations of milk double-stranded DNA were used to normalise the samples 250 ng/μL of DNA and samples were pooled accordingly. For samples with very low DNA concentration, the maximum 10 μL of sample was pooled. Based on previous bovine milk mycobiome work, we anticipated that a subset of samples would be negative for fungi [13]. Therefore, we pooled samples into three pools based on DNA concentration and PCR amplification (high, intermediate, and low DNA concentration). Pooled DNA was then cleaned using DNA Clean & Concentrator (Zymo Research, USA) and the concentration was measured using Qubit dsDNA HS Assay Kit (Invitrogen, CA, USA). To minimise the potential impact of well-to-well contamination for low DNA samples [52], the final pooled DNA consisted of high, intermediate, and low DNA with proportions of 80:10:10.

### Fungal sequencing processing

Overlapping paired-end reads were processed with DADA2 pipeline [53] using the QIIME 2 v.2018.6 [54]. Unique amplicon sequence variants (ASVs) were assigned a taxonomy and aligned to the 2017 release of the UNITE v.7 reference database at 99% sequence similarity [55]. Demultiplexed sequencing data was deposited into the Sequence Read Archive of NCBI (accession number PRJNA536254).

Initial preprocessing of ASVs was conducted using Phyloseq v. 1.26.1 [56]. Overall, 1421 unique ASVs were detected. ASVs belonging to kingdoms other than Fungi (e.g. plants, *n* = 334 ASVs) were removed. We obtained a mean (SD) of 16,520 (78,489) and median (IQR) of 52 (13–709) high quality fungal sequencing reads per milk sample, compared with 129,100 (69,044) reads from the positive controls (stool sample and mock community), and 9 (17) reads in negative controls. The mock community was dominated by *Cryptococcus neoformans* (Basidiomycota phylum) suggesting sequencing bias against Ascomycota. We did not identify any fungal contaminant ASVs using decontam [57]. The majority of samples had very low sequencing reads with 184/271 having less than 300 reads per sample. The threshold applied to define presence of fungi was informed by the presence of visible PCR bands on gel electrophoresis. There was a clear trend between the presence of a PCR band and the number of sequencing reads, with 9% of samples containing 300–500 reads having a band vs. 71% of samples with 1000–5000 and 91% of samples with more than 5000 reads (Fig. 1a). To optimize the retention of samples that were PCR-positive by visual inspection, as well as the elimination of samples with very low sequencing depth, samples with at least 1000 sequencing reads were considered positive for fungi. These samples were rarefied to the minimum sequencing depth of 1000 sequences, resulting in 58 fungi-positive samples containing a total of 625 fungal ASVs. The number of reads for each ASV was relativized to the total sum. Fungal richness (observed taxa) and diversity (inverse Simpson index) were estimated at ASV and genus levels.

### Covariates

Participants were recruited from four Canadian cities: Vancouver (49.2827° N, 123.1207° W, British Columbia), Edmonton (53.5444° N, 113.4909° W, Alberta), Manitoba (49.8951° N, 97.1384° W, Winnipeg and two rural towns), and Toronto (43.6532° N, 79.3832° W, Ontario). Average climate information from 1981 to 2010 was obtained from Environment and Climate Change Canada (http://climate.weather.gc.ca; access date 13 March 2019; used in descriptive but not statistical analyses). Infant sex, birth weight, gestational age, and birth mode were documented from hospital records. Infant feeding, history of oral thrush, and season of milk sample collection were reported by standardized questionnaire. At the time of sample collection (3–4 months), breastfeeding status was classified as “exclusive” (human milk only) or “partial” (human milk supplemented with infant formula or supplementary food). The mode of breastmilk feeding was also reported and classified as “all direct breastfeeding” (nursing at the breast only, with no feeding of pumped milk), or “some pumped milk” (at least one serving of pumped milk in the past 2 weeks) [58]. Population density was estimated for CHILD participants’ homes using 2006 census data on population counts within the smallest available census geographical boundary (250 m) and was categorized as high or low (above or below the median). Census data also provided the rural vs. urban residence. Green space exposure was quantified using the derived normalized difference vegetation index (NDVI) in a 250 m buffer around the mother’s residential addresses. NDVI, with values ranging from − 1 to 1 is an indicator of overall greenness based on land surface reflectance of visible and near infrared parts of spectrum. Time weighted averages across the 12 months postpartum were assigned and categorized into grey (− 1.0 to 0.2), moderate green (0.2 to 0.3), and green (0.3 to 1.0). Home environment characteristics including dust, moisture, and visible mould levels were determined based on questionnaires completed by mothers and a walk-through home assessment by trained research staff from the CHILD Study [59]; each exposure was categorized into high or low (above or below median). Bacterial V4 16S rRNA gene sequencing was previously performed [24].

### Statistical analysis

Data analysis was conducted in R v. 3.5.2 [60]. Presence/absence was tested by κ2 and logistic regression for categorical variables and analysis of variance (ANOVA) for continuous variables. Bacterial enrichment based on presence of fungi was assessed by linear discriminant analysis effect size (LEfSe) with default parameters and logarithmic LDA score threshold of 4 [33]. Association of fungal structure with covariates was assessed using ANOVA after centre log-ratio transformation [61, 62]. *P* values were corrected with Benjamini-Hochberg’s false discovery rate (FDR) method [63]. Milk microbiota outliers were defined as those contributing greater than the median plus twice the interquartile range of the sample variance to the total [31]. LASSO was performed using glmnet package with default parameters to define the optimum lambda with 10-fold cross-validation. Using the optimum lambda, coefficients were estimated for model parameters [64]. While LASSO does not necessarily provide evidence for biological associations, it is a good technique for feature selection as it shrinks the beta coefficients of variables with minimal contribution to zero. This approach minimally increases the bias while enhancing the interpretability of results. LASSO is most suitable when a small to moderate number of moderate-sized effects are expected [65] (as is the case in our study). Association of LASSO-selected variables with fungal presence was assessed using multivariable logistic regression and the performance was assessed by area under the receiver operation curve (AUC). AUC values correspond to the accuracy of a binary classification with higher numbers indicative of higher accuracy [66]. Association of fungal alpha diversity with host and environmental factors were assessed by Wilcoxon rank sum or Kruskal-Wallis tests. Correlation of bacterial and fungal alpha diversity measures was assessed using Spearman rank correlation following log10 transformation. Fungi β diversity was assessed on Bray-Curtis dissimilarity using permutation ANOVA (PERMANOVA). Data analyses codes will be available upon request.

### Inter-kingdom network analysis

Co-occurrence of the most abundant bacterial and fungal genera (> 1% mean relative abundance) were assessed by Spearman rank correlation using CoNet [67]. One hundred bootstrap samples were used to infer pseudo *p* values. Only edges with correlation scores of > 0.5 and *p* < 0.05 (Bonferroni-corrected) were retained. The network was visualised in Cytoscape [68].

## Supplementary information

**Additional file 1: Table S1.** Characteristics of mother-infant dyads from the CHILD cohort included in this analysis (*N* = 271) in comparison with previous bacterial analysis (*N* = 393). **Table S2.** Milk characteristics of mother-infant dyads for fungal sequencing. **Table S3.** Comparison of characteristics of mother-infant dyads from the CHILD cohort included in this study across study cities. **Table S4.** Most abundant fungal genera (> 1% mean relative abundance) in the human milk microbiota among 58 mothers with detectable milk fungi in the CHILD cohort. **Figure S1.** Milk bacterial composition outlier. **Figure S2.** Association of milk bacterial clusters and bacterial composition outliers with fungal richness and diversity tested by ANOVA.

## Data Availability

The datasets generated and analysed during the current study are available in the Sequence Read Archive of NCBI repository (BioProject accession number PRJNA536254, https://www.ncbi.nlm.nih.gov/bioproject/?term=PRJNA536254).

## Abbreviation

ASV: Amplicon sequencing variant
AUC: Area under the curve
BMI: Body mass index
CI: Confidence interval
dsDNA: Double stranded DNA
DSLNT: Disialyllacto-N-tetraose
FDR: False discovery rate
FUT2: Fucosyl transferase 2
HMO: Human milk oligosaccharide
ITS: Internal transcribed spacer
Km: Kilometer
LASSO: Least absolute shrinkage and selection operator
LefSe: Linear discriminant analysis effect size
LNH: Lacto-N-hexaose
NDVI: Normalized difference vegetation index
OR: Odds ratio
PCR: Polymerase chain reaction
PERMANOVA: Permutation ANOVA
SD: Standard deviation
