# Supplementary material for: Human milk fungi: environmental determinants and inter-kingdom associations with milk bacteria in the CHILD Cohort Study
Source: BMC Microbiol. 2020 Jun 5;20:146. doi: 10.1186/s12866-020-01829-0 (PMC7275434; doi:10.1186/s12866-020-01829-0)
Supplement: Supplementary file 1 — Additional file 1: Table S1. Characteristics of mother-infant dyads from the CHILD cohort included in this analysis (N = 271) in comparison with previous bacterial analysis (N = 393). Table S2. Milk characteristics of mother-infant dyads for fungal sequencing. Table S3. Comparison of characteristics of mother-infant dyads from the CHILD cohort included in this study across study cities. Table S4. Most abundant fungal genera (> 1% mean relative abundance) in the human milk microbiota among 58 mothers with detectable milk fungi in the CHILD cohort. Figure S1. Milk bacterial composition outlier. Figure S2. Association of milk bacterial clusters and bacterial composition outliers with fungal richness and diversity tested by ANOVA. [file 12866_2020_1829_MOESM1_ESM.pdf]

Human milk fungi: environmental determinants and inter-kingdom associations with milk bacteria in CHILD Cohort Study (Moossavi et. al.)

Supplementary Tables and Figures:

Table S1. Characteristics of mother-infant dyads from the CHILD Cohort included in this analysis (N=271) in comparison with previous bacterial analysis (N=393).

Table S2. Milk characteristics of mother-infant dyads for fungal sequencing.

Table S3. Comparison of characteristics of mother-infant dyads from the CHILD Cohort included in this study across study cities

Table S4. Most abundant fungal genera (>1% mean relative abundance) in the human milk microbiota among 58 mothers with detectable milk fungi in the CHILD Cohort.

Figure S1. Milk bacterial composition outlier.

Figure S2. Association of milk bacterial clusters and bacterial composition outliers with fungal richness and diversity tested by ANOVA.

Supplementary references

Table S1. Characteristics of mother-infant dyads from the CHILD Cohort included in this analysis (N=271) in comparison with previous bacterial analysis (N=393).

	Characteristics	Mean ± SD or n (%)[a]	
		Fungal study (N=271)	Bacterial study (N=393)
Maternal	FUT2 secretor	195 (72.8)	279 (71.7)
	Ethnicity		
	Caucasian	197 (72.7)	287 (73.0)
	Asian	52 (19.2)	73 (18.6)
	First Nations	10 (3.7)	15 (3.8)
	Other	12 (4.2)	18 (4.6)
	Prenatal smoking	13 (4.8)	18 (4.6)
	History of atopy	181 (67)	251 (63.9)
Infant	Birth weight (g)	3476 ± 476	3469 ± 469
	Female sex	125 (46.1)	192 (48.3)
	Gestational age (weeks)	39.2 ± 1.3	39.2 ± 1.3
	History of oral thrush	33 (12.2)	46 (11.8)
	Child antibiotics before 3-4 months	9 (3.3)	11 (2.9)
	Asthma at 3 years [b]	48 (17.7)	47 (12.5)
Early life	Mode of delivery		
	Elective C/S	34 (12.6)	46 (11.9)
	Emergency C/S	32 (11.9)	47 (12.1)
	Vaginal	203 (75.5)	294 (76.0)
	Older siblings		
	None	148 (54.6)	211 (53.7)
	One	89 (32.8)	126 (32.1)
	Two or more	34 (12.5)	56 (14.2)
	Maternal intrapartum antibiotics	96 (35.6)	131 (35.4)
BF	Direct BF (at the breast) only	108 (40.0)	162 (41.9)
	Pump expression [c]	77 (81.9)	91 (81.2)
Home environment	Sample collection season		
	Spring (March-May)	58 (21.5)	72 (18.6)
	Summer (June-August)	64 (27.4)	103 (26.5)
	Autumn (September-November)	74 (23.7)	112 (28.9)
	Winter (December-February)	74 (27.4)	101 (26.0)
	High mould [d]	90 (33.3)	137 (34.9)
	High moisture [d]	120 (44.4)	183 (46.6)
	High dust [d]	144 (53.3)	208 (52.9)
	Dog ownership	82 (30.2)	119 (30.2)
	Furry pets	137 (50.6)	201 (51.0)
	Urban residence	242 (95.7)	91 (92.8)
	High population density	126 (49.8)	195 (52.7)
	Residential vegetation during the 1st year [e]		
	Grey	16 (6.0)	20 (5%)
	Moderately green	92 (34.3)	135 (35%)
	Green	160 (59.7)	233 (60%)
	Study sites		
	Vancouver	67 (24.7)	96 (24.3)
	Edmonton	61 (22.5)	99 (25.1)
	Manitoba	81 (29.9)	96 (24.3)
	Toronto	62 (22.9)	104 (26.3)

[a] Percentages are calculated after excluding dyads with missing data. BF, breastfeeding; BMI, body mass index; C/S, Caesarean section; HMO, human milk oligosaccharide. [b] Asthma was diagnosed by an expert study physician at the at age 3 years and classified for this analysis as "possible or probable asthma" or "no asthma". [c] data available for a subset of dyads (n=94); [d] Each exposure was categorized into high or low (above or below median); [e] Defined based on normalized difference vegetation index (NDVI) in a 250 m buffer around the mother's residential addresses during the

Table S2. Milk characteristics of mother-infant dyads for fungal sequencing.

Characteristics	Current Fungal study (N=271)	Previous Bacterial study (N=393)
Bacterial richness (observed ASVs)		
Overall	145.3 ± 46.3	146.8 ± 43.8
Proteobacteria	104 ± 29.9	105.9 ± 28.4
Firmicutes	16.4 ± 15.1	15.9 ± 13.6
Actinobacteria	14.0 ± 5.6	14.1 ± 5.4
Bacterial diversity (Inverse Simpson Index)		
Overall	15.2 ± 9.2	15.8 ± 8.9
Proteobacteria	16.3 ± 7.5	16.8 ± 6.9
Firmicutes	3.2 ± 1.5	3.2 ± 1.4
Actinobacteria	3.8 ± 1.8	3.7 ± 1.7
Bacterial taxonomic clusters [a]		
C1	33 (12.2)	42 (10.7)
C2	73 (27.0)	98 (24.9)
C3	93 (34.5)	161 (41.0)
C4	71 (26.3)	92 (23.4)
Bacterial composition outliers [b]	35 (12.9)	45 (11.5)
HMO diversity	4.9 ± 1.4	4.9 ± 1.4
HMO concentration (mg/mL)	10.2 ± 2.1	10.2 ± 2.1

[a] Defined based on hierarchical clustering of the core ASVs as previously described [1]. [b] Defined as those contributing greater than the median plus twice the interquartile range of the sample variance to the total [2]. All dyads included in the current fungal study were also included in the previous bacterial study [1].

Table S3. Comparison of characteristics of mother-infant dyads from the CHILD Cohort included in this study across study cities

Characteristics	Mean ± SD or n (%)[a]				P value
	Vancouver	Edmonton	Manitoba	Toronto	
Maternal, infant, and early life					
Birth mode<br>Vaginal<br>Elective C/S<br>Emergency C/S	 53 (79.1)<br>6 (9.0)<br>8 (11.9)	 44 (72.1)<br>9 (14.8)<br>8 (13.1)	 63 (79.8)<br>8 (10.1)<br>8 (10.1)	 43 (69.4)<br>11 (17.7)<br>8 (12.9)	0.723
Maternal atopy	44 (65.7)	34 (55.7)	60 (74.1)	43 (69.4)	0.137
Female sex	31 (46.3)	30 (49.2)	36 (44.5)	28 (45.2)	0.951
Multiparous	21 (31.3)	28 (45.9)	46 (56.7)	28 (45.2)	**0.022**
Breastfeeding and HMOs					
Direct BF (at the breast) only	27 (40.3)	27 (44.3)	29 (36.3)	25 (40.3)	0.816
Exclusive BF at sample collection	41 (61.2)	21 (34.4)	42 (51.9)	26 (41.9)	**0.014**
Total HMO concentration (mg/mL)	10.3+2.3	10.5+2.1	10.0+2.2	10.1+1.8	0.505
DSLNT (%)	1.46+1.61	2.23+1.47	1.82+1.85	1.98+1.83	0.074
LNH (%)	0.53+0.41	0.54+0.43	0.67+0.55	0.49+0.39	0.11
Home environment					
Sample collection season<br>Spring<br>Summer<br>Autumn<br>Winter	 9 (13.6)<br>14 (21.2)<br>20 (30.3)<br>23 (34.8)	 17 (27.9)<br>13 (21.3)<br>13 (20.9)<br>18 (29.5)	 21 (25.9)<br>14 (17.3)<br>28 (34.6)<br>18 (22.2)	 11 (17.7)<br>23 (37.1)<br>13 (21.0)<br>15 (24.2)	0.056
High mould	29 (43.3)	15 (25.0)	12 (14.8)	34 (55.7)	**< 0.001**
High moisture	29 (43.3)	29 (47.5)	41 (50.6)	21 (34.4)	0.261
High dust	41 (61.2)	42 (68.8)	32 (39.5)	29 (47.5)	**0.002**
Furry pets ownership	28 (41.8)	38 (62.3)	43 (53.1)	28 (45.2)	0.095
Urban residence	54 (98.1)	58 (98.3)	71 (89.9)	59 (98.3)	**0.026**
High population density	41 (74.5)	15 (25.4)	23 (29.1)	47 (78.3)	**< 0.001**
Residential vegetation during the 1st year [b]<br>Grey<br>Moderately green<br>Green	 2 (3.0)<br>11 (16.7)<br>53 (80.3)	 4 (6.7)<br>33 (55.0)<br>23 (38.3)	 6 (7.4)<br>41 (50.6)<br>34 (42.0)	 4 (6.6)<br>7 (11.5)<br>50 (81.9)	**< 0.001**
Climate [c]					
Daily average temperature (°C)	10.4	4.2	3.0	8.2	-
Daily maximum temperature (°C)	13.9	9.3	8.7	13.0	-
Daily minimum temperature (°C)	6.8	-1.0	-2.7	3.3	-
Annual precipitation (mm)	1189	456	521	786	-
Days with ≥5mm precipitation	77	25	29	49	-
Average Relative Humidity - 0600LST (%)	85.9	76.3	82.4	81.3	-
Average Relative Humidity - 1500LST (%)	70.3	54.1	61.1	61.3	-
Landscape	Coastal	Prairie	Prairie	Shield	-

[a] Percentages are calculated after excluding dyads with missing data. Other factors tested but not shown: maternal ethnicity, intrapartum antibiotics, dog ownership, maternal secretor status, and maternal prenatal smoking.
BF, breastfeeding; BMI, body mass index; C/S, caesarean section; HMO, human milk oligosaccharide.
[b] Defined based on normalized difference vegetation index (NDVI) in a 250 m buffer around the mother's residential addresses during the 12 months postpartum
[c] From results of 1981-2019 http://climate.weather.gc.ca/climate_normals (accessed 13 March 2019)

Table S4. Most abundant fungal genera (>1% mean relative abundance) in the human milk microbiota among 58 mothers with detectable milk fungi in the CHILD Cohort.

Phylum	Genera	Prevalence (%)[a]	Relative abundance (%)		
			Mean±SD	CV	Maximum
Ascomycota	*Candida*	60.3	28.6±38.2	1.3	100
Ascomycota	*Clavispora*	3.4	2.7±14.5	5.3	79.1
Ascomycota	*Exophiala*	17.2	3.2±15.3	4.8	90.7
Ascomycota	*Penicillium*	3.4	2.8±15.3	5.5	98.8
Ascomycota	*Alternaria*	50.0	6.9±20.8	3.0	94.5
Ascomycota	Unclassified *Capnodiales*	20.7	2.8±10.4	3.8	56.7
Ascomycota	*Aureobasidium*	12.1	2.7±13.6	4.9	82.1
Ascomycota	Unclassified *Dipodascus*	1.7	1.5±11.2	7.6	85.3
Ascomycota	Unclassified *Trichomeriaceae*	5.2	1.1±8.0	7.6	61.1
Basidiomycota	*Filobasidium*	3.4	1.7±12.4	7.5	94.6
Basidiomycota	*Rhodotorula*	43.1	10.3±24.7	2.4	96.9
Basidiomycota	*Malassezia*	6.9	1.0±7.3	7.1	55.5

[a] Percentage of samples where the taxa was present. This analysis is restricted to the 58 samples (of 271 analysed) where fungi were detected.
CV, coefficient of variation.

Figure S1. Milk bacterial composition outlier. A) Identification based on Principal Component (PC)1 variance. B) Beta diversity of milk bacterial composition based on Bray-Curtis dissimilarity measure.

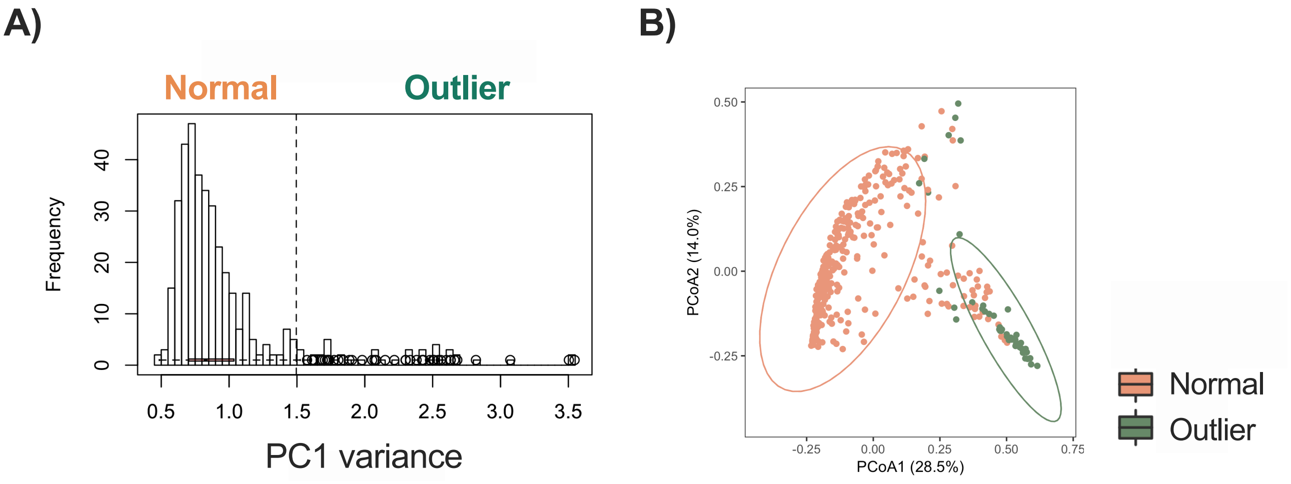

Figure S2. Association of milk bacterial clusters and bacterial composition outliers with fungal richness and diversity tested by Kruskal-Wallis and Wilcoxon rank sum tests, respectively.

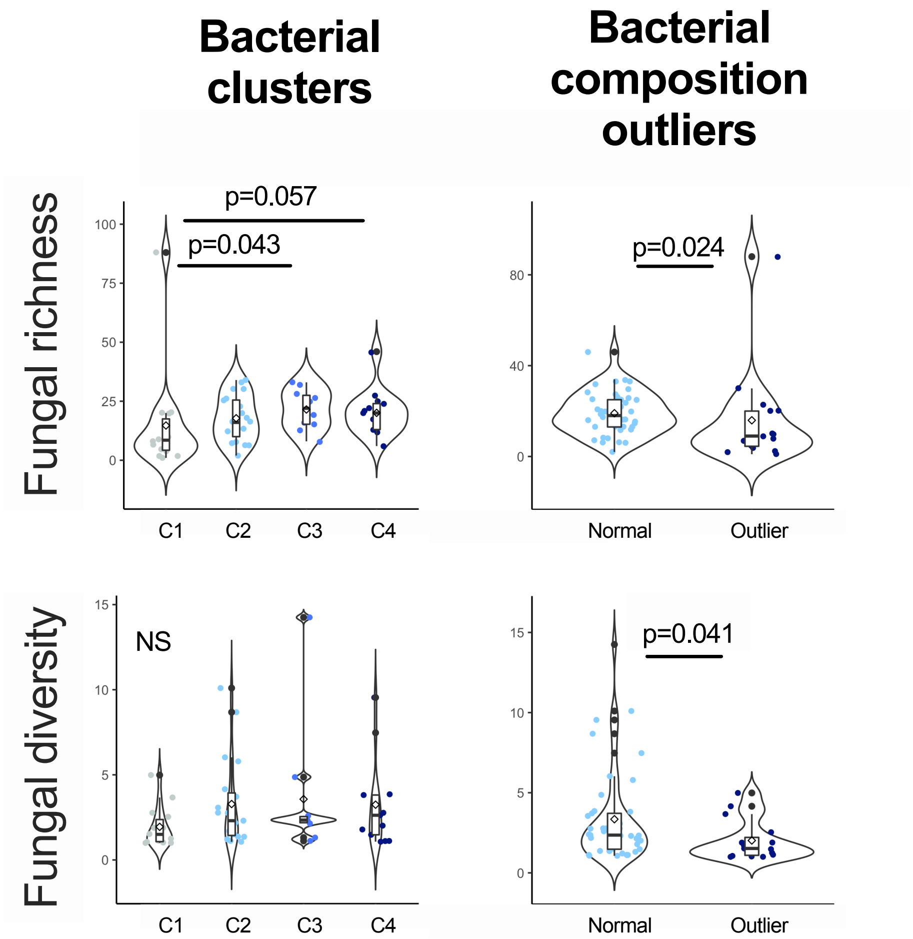

Supplementary references

1. Moossavi S, Sepehri S, Robertson B, Bode L, Goruk S, Field CJ, Lix LM, de Souza RJ, Becker AB, Mandhane PJ *et al*: Composition and Variation of the Human Milk Microbiome is Influenced by Maternal and Early Life Factors *Cell Host Microbe* 2019, 25:324-335.

2. Gloor GB, Wu JR, Pawlowsky-Glahn V, Egozcue JJ: It's all relative: analyzing microbiome data as compositions. *Ann Epidemiol* 2016, 26(5):322-329.
